# Aligning Cancer Research Priorities in Europe with Recommendations for Conquering Cancer: A Comprehensive Analysis

**DOI:** 10.3390/healthcare12020259

**Published:** 2024-01-19

**Authors:** Denis Horgan, Marc Van den Bulcke, Umberto Malapelle, Nicola Normanno, Ettore D. Capoluongo, Arsela Prelaj, Carmelo Rizzari, Aliki Stathopoulou, Jaya Singh, Marta Kozaric, France Dube, Manuel Ottaviano, Stefania Boccia, Gabriella Pravettoni, Ivana Cattaneo, Núria Malats, Reinhard Buettner, Karim Lekadir, Francesco de Lorenzo, Patricia Blanc, Catherine Alix-Panabieres, Sara Badreh, Paul Hofman, Eric Solary, Ruggero De Maria

**Affiliations:** 1 European Alliance for Personalised Medicine, 1040 Brussels, Belgium; jayasinghtec29@gmail.com (J.S.); marta.kozaric@euapm.eu (M.K.); 2 Department of Molecular and Cellular Engineering, Jacob Institute of Biotechnology and Bioengineering, Faculty of Engineering and Technology, Sam Higginbottom University of Agriculture, Technology and Sciences, Prayagraj 211007, India; 3 Belgian Cancer Centre, Sciensano, 1050 Brussels, Belgium; marc.vandenbulcke@sciensano.be; 4 Department of Public Health, University Federico II of Naples, 80138 Naples, Italy; umberto.malapelle@unina.it; 5 Istituto Nazionale Tumori “Fondazione G. Pascale”—IRCCS, 80131 Naples, Italy; n.normanno@istitutotumori.na.it; 6 Dipartimento di Eccellenza in Medicina Molecolare e Biotecnologie Mediche, Università Federico II, 80138 Naples, Italy; edotto70@gmail.com; 7 Department of Clinical Pathology and Genomics, Azienda Ospedaliera Per L’Emergenza Cannizzaro, 95126 Catania, Italy; 8 Department of Medical Oncology, Fondazione IRCCS Istituto Nazionale Tumori, 20133 Milan, Italy; arsela.prelaj@istitutotumori.mi.it; 9 Unità di Ematologia Pediatrica, Fondazione MBBM, Università di Milano-Bicocca, 20126 Monza, Italy; carmelo.rizzari@gmail.com; 10 European Cancer Patient Coalition, 1000 Brussels, Belgium; aliki.stathopoulou@ecpc.org (A.S.); francesco.delorenzo@ecpc.org (F.d.L.); 11 Astra Zeneca, Concord Pike, Wilmington, DE 19803, USA; france.dube@astrazeneca.com; 12 Departamento de Tecnología Fotónica y Bioingeniería, Universidad Politècnica de Madrid, 28040 Madrid, Spain; manuel.ottaviano@upm.es; 13 Section of Hygiene, Department of Life Sciences and Public Health, Università Cattolica del Sacro Cuore, 20123 Rome, Italy; stefania.boccia@unicatt.it; 14 Departments of Woman and Child Health and Public Health, Fondazione Policlinico Universitario A. Gemelli IRCCS, 00168 Rome, Italy; 15 Department of Oncology and Hemato-Oncology, University of Milan, 20122 Milan, Italy; gabriella.pravettoni@ieo.it; 16 Applied Research Division for Cognitive and Psychological Science, European Institute of Oncology (IEO) IRCCS, 20139 Milan, Italy; 17 Novartis Farma SpA, 20154 Milano, Italy; ivana.cattaneo@novartis.com; 18 Genetic and Molecular Epidemiology Group, Spanish National Cancer Research Centre (CNIO), 28029 Madrid, Spain; nmalats@cnio.es; 19 Lung Cancer Group Cologne, Institute of Pathology and Medical Faculty, Center for Integrated Oncology Cologne/Bonn, University Hospital Cologne, 50937 Cologne, Germany; reinhard.buettner@uk-koeln.de; 20 Artificial Intelligence in Medicine Lab (BCN-AIM), Universitat de Barcelona, 08007 Barcelona, Spain; karim.lekadir@ub.edu; 21 Imagine for Margo, 78100 Saint-Germain-en-Laye, France; patricia.blanc@imagineformargo.org; 22 Laboratory of Rare Human Circulating Cells, University Medical Center of Montpellier, 34093 Montpellier, France; c-panabieres@chu-montpellier.fr; 23 Cancer Childhood International, 1200 Vienna, Austria; sara@bwconsultancy.se; 24 Laboratory of Clinical and Experimental Pathology, Pasteur Hospital, Université Côte d’Azur, 06000 Nice, France; hofman.p@chu-nice.fr; 25 INSERM U1287, Gustave Roussy Cancer Campus, 94805 Villejuif, France; eric.solary@gustaveroussy.fr; 26 Faculty of Medicine, Université Paris-Sud, Le Kremlin-Bicêtre, 91405 Île-de-France, France; 27 Department of Hematology, Gustave Roussy Cancer Campus, 94805 Paris, France; 28 Institute of General Pathology, Catholic University of the Sacred Heart, 20123 Rome, Italy; presidenza@alleanzacontroilcancro.it

**Keywords:** cancer, survey, patients, research, priorities, policymakers, recommendations, personalized medicine, reimbursement

## Abstract

Improvements in cancer care require a new degree of collaboration beyond the purely medical sphere, extending deeply into the world of other stakeholders—preeminently patients but also the other stakeholders in the hardware and software of care. Cancer remains a global health challenge, necessitating collaborative efforts to understand, prevent, and treat this complex disease. To achieve this goal, a comprehensive analysis was conducted, aligning the prioritization of cancer research measures in 13 European countries with 13 key recommendations for conquering cancer in the region. The study utilized a survey involving both patients and citizens, alongside data from IQVIA, a global healthcare data provider, to assess the availability and access to single-biomarker tests in multiple European countries. The results revealed a focused approach toward understanding, preventing, and treating cancer, with each country emphasizing specific research measures tailored to its strengths and healthcare objectives. This analysis highlights the intricate relationship between research priorities, access to biomarker tests, and financial support. Timely access to tests and increased availability positively influence research areas such as cancer prevention, early detection, ageing, and data utilization. The alignment of these country-specific measures with 13 recommendations for conquering cancer in Europe underscores the importance of tailored strategies for understanding, preventing, and treating cancer.

## 1. Introduction

Cancer, a disease that can be effectively prevented, detected, diagnosed, and treated, benefits from advances in understanding its biological processes, risk factors, and health determinants. Early diagnosis and advancements in treatments have significantly improved the lives of cancer patients and survivors [1,2]. However, addressing the challenges of cancer requires collaboration among stakeholders at both the national and European Union (EU) levels in Europe. Despite progress, many cancer cases are diagnosed at an advanced stage, presenting challenges as they can be highly aggressive and resistant to current therapies. Europe’s Beating Cancer Plan, initiated in 2021, aims to leverage new technologies and scientific advancements, including insights from comorbidities and the social and behavioral sciences to enhance cancer care throughout the disease pathway [3,4,5].

Private health insurance, contributing approximately 10% of all health spending across the Organization for Economic Cooperation and Development (OECD) countries, plays a vital role in health financing after government schemes, social health insurance, and out-of-pocket payments. However, its contribution varies significantly among the OECD countries. On average, it finances one in every ten U.S. dollars (USD) spent on health across the OECD. Notably, private health insurance plays a significant role in the United States (33% of health spending), nearly half in Switzerland, and approximately 60% in the Netherlands. In contrast, its role is minimal in countries such as the Czech Republic, Estonia, and Sweden, where it accounts for 5% or less of health spending [6]. Voluntary private health insurance, which generally provides a more limited range of services, represents 11–14% of overall health spending in Slovenia, Canada, Australia, Ireland, and Israel [6]. Universal health coverage (UHC) is present in all European countries, offering valuable insights for global efforts to advance health for all and manage the private sector within health systems. 

**Figure 1:**
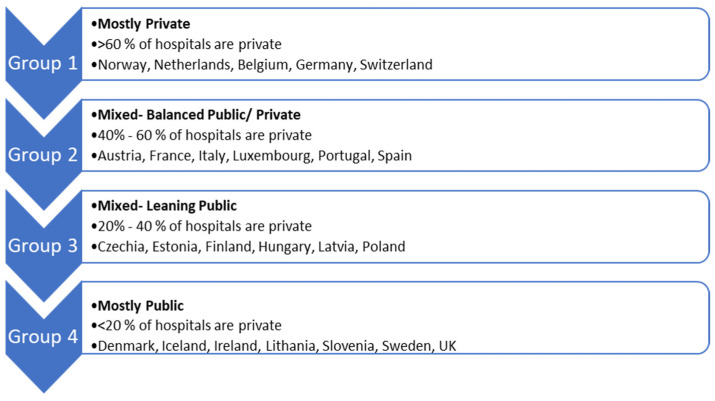
Different models within European health systems [7].

There are four healthcare provision models, with the private sector playing a predominant role in some countries and a minimal role in others (Figure 1). Hospital structures are evolving in Europe, with outpatient services shifting outside medical facilities, and countries striving for increased efficiency. Private hospitals vary in their bed capacities across Europe, providing beds in proportion to their importance in some countries and focusing solely on outpatient care in others. Primary health care is mostly public in Iceland, Slovenia, Denmark, the UK, Ireland, and Sweden. Countries with public national reimbursement processes tend to have better access to medicines [7,8].

The landscape of clinical trials in oncology has experienced considerable transformation, driven by advancements in identifying biomarkers and their alignment with therapies. In 2018, a substantial 55% of oncology clinical trials incorporated biomarkers, marking a significant increase from the 15% recorded in 2000 [9,10]. This progression anticipates the establishment of comprehensive biomarker testing, leading to a shift away from traditional organ-of-origin focused treatments to tumor-agnostic treatments based on molecular features [11]. The efficient implementation of biomarker testing, facilitated by novel technologies, stands as a focal point in Europe’s Beating Cancer Plan Flagship 6 [4,12].

There has been a notable shift away from the conventional determination of research agendas by researchers, institutions, and funding organizations, with the corporate sector demonstrating the benefits of involving stakeholders. This movement aligns with a broader trend globally, endorsed by influential entities such as the European Commission (EC), the Organization for Economic Cooperation and Development (OECD), the World Health Organization (WHO), and the UK’s National Institute for Health Research (NIHR) advocating for the active engagement of non-research stakeholders in agenda setting [13].

Europe’s Beating Cancer Plan, formulated through extensive national and international consultations, explicitly recognizes the value of partnerships with its ‘Health in All Policies’ multistakeholder approach. This approach incorporates the perspectives of stakeholder groups, patients, the European Parliament, and Member States, addressing the entire spectrum of cancer, from prevention to early detection, diagnosis, treatment, and the quality of life of cancer patients and survivors [4]. It is noteworthy that the European Commission’s major pharmaceutical reform involved consultations with a range of “key” stakeholders, including organizations representing patients, consumers, civil society, health care professionals, healthcare providers, researchers, academia, learned societies, and the pharmaceutical industry [14].

Breaking down barriers at both policy-making and operational levels is crucial. While primary care prevention is widely recommended, delivering a variety of relevant services is often unfeasible for most clinicians, especially in low-resource and rural healthcare settings. The gap between research and practical implementation in cancer prevention and control, as in other preventive healthcare areas, is well-documented [15]. A study reports an average of 17 years for only 14% of research evidence to be put into practice. The clinical burden of implementation is significant, particularly for evidence-based practices like lung cancer screening, in the absence of clear guidelines or unfamiliar processes. Collaboration between clinicians and researchers and tailoring services to meet primary care partners’ preferences is complex, requiring understanding specific needs and priorities. This understanding is essential for preventive medicine researchers, program planners, and funders to comprehend commonalities and distinctions, especially when comparing rural and non-rural practices and other clinician and practice characteristics [16]. Establishing a standardized approach is crucial for comparing research priorities across different diseases or health topics, using methods such as the research cycle type framework consistently applied to distinct topics in public health. However, even at WHO headquarters, there is variation in the utilization of research priority-setting methodologies [17].

### Conquering Cancer: A Mission within Reach

Accurate diagnosis and the development of effective treatments for currently untreatable or intractable cancers hinge on a comprehensive understanding of the biological processes within human cells. This is particularly crucial concerning childhood cancers, adolescent and young adult cancers, and cancers in the elderly population, given their unique biological and clinical characteristics. A deep comprehension of cancer complexity, encompassing factors like lifestyle, environment, workplace exposures, sex/gender, and age, is essential for crafting effective preventive measures. Furthermore, there is an urgent need for a better understanding of the impact of cancer treatment on patients to optimize their care and enhance their quality of life. In collaboration with citizens, patients, and stakeholders from member states, the EU’s Cancer Mission board has formulated 13 specific recommendations to comprehensively address cancer. The recommendations cover understanding cancer, its risk factors, and impacts, preventing avoidable cases, optimizing diagnosis and treatment, and supporting the quality of life for cancer survivors while ensuring equal access for all. The recommendations, detailed in Table 1, collectively contribute to a comprehensive and inclusive approach to conquering cancer, addressing its distinct facets, and ensuring equal access to quality care for all [18].

The objective of the study is to understand and address the challenges in cancer prevention and control, as well as advocate for a holistic approach to conquer cancer.

## 2. Materials and Methods

The European Cancer Patient Coalition (ECPC) and Childhood Cancer International-Europe (CCI-E) carried out a survey involving both patients and citizens. Subsequently, the European Alliance for Personalized Medicine (EAPM) conducted an in-depth analysis of the survey findings to identify associations and trends in cancer research priorities. This survey encompassed breast cancer, prostate cancer, lung cancer, colon cancer, and other gastrointestinal cancers. Breast, prostate, lung, colon, and gastrointestinal cancers are among the most prevalent and impactful types of cancer globally. They affect a large number of individuals and have significant public health implications. These types of cancers are of particular interest also due to their relevance to healthcare policies, screening programs, and other public health initiatives. The survey garnered responses from participants across 30 countries, but this study focuses on 13 countries: Belgium, Bulgaria, France, Italy, Germany, Spain, Slovakia, Romania, Portugal, Netherlands, Luxembourg, Hungary, and Greece. By focusing on a smaller group of countries, it becomes more feasible to delve deeper into the details and nuances of each country’s situation, facilitating meaningful comparisons. Moreover, the chosen countries cover all the main European regions. The study also sourced data from IQVIA, a global healthcare data provider, to assess single biomarker test availability and access in multiple European countries.

### 2.1. A Survey of Patients and Citizens

The patient and citizen survey (provided as Supplementary Materials), designed by the European Cancer Patient Coalition (ECPC) and Childhood Cancer International-Europe (CCI-E), is a part of the broader “UNCAN.eu” initiative, which represents a collaborative European endeavor aimed at identifying cancer research priorities and the expectations of patients and the general public. The ultimate goal is to incorporate these perspectives into the formulation of research and innovation endeavors. The survey was carried out as part of a coordination and support action that is laying the groundwork for establishing a European Cancer Research Data Hub, which will pinpoint cancer research priorities where there is an overlap between the expectations of patients, the general public, and the scientific community [19,20]. This online survey included over 1700 participants and it was disseminated through various channels, including professional, social, and scientific networks such as websites and social media. The data collection took place from 20 November 2022 to 20 February 2023. It encompassed adult cancer patients, cancer survivors, caregivers, pediatric cancer patients, and individuals not directly impacted by cancer. It assessed different aspects of cancer research using 35 measures derived from the six foundational pillars of research. The responses of the 16–70+ age group were analyzed by the EAPM, and the results focusing on breast, prostate, lung, colon, and other gastrointestinal cancers were compared in detail across 13 countries (Belgium, Bulgaria, France, Italy, Germany, Spain, Slovakia, Romania, Portugal, Netherlands, Luxembourg).

(a) Priority Areas for Cancer Research

Cancer research areas and subtopics among respondents were analyzed to inform European Commission (EC) prioritization in terms of policies, budgets, and resources. The research areas are ranked based on the frequency of mentions and perceived significance.

(b) Differences in Priorities Based on Cancer Type

The priorities of respondents with different types of breast, lung, prostate, and other cancers were compared, noting variations in the importance they attached to specific research areas. The questionnaire covered six research topics previously identified by the expert working groups from the European Cancer Patient Coalition (ECPC) and the Childhood Cancer International Europe (CCI-E).

Six pillars were identified:

- Factors influencing cancer development and risk;
- Cancer prevention and early detection;
- Cancer biology and therapeutic approaches;
- Aging and its intersections with cancer;
- Cancer complications and survivorship;
- Data generation and utilization in cancer research.

Under these six pillars, 35 measures were identified (Table 2).

### 2.2. IQVIA Data on Single Biomarkers

The data for this study are sourced from IQVIA (standing for “I” for “Information,” “Q” for “Quality,” “V” for “Value,” “I” for “Insight,” and “A” for “Advancing”) which has provided comprehensive data on availability and access to single-biomarker tests in multiple European countries.

Data Parameters:

1. Single-Biomarker Test Access: This parameter measures the average proportion of laboratories offering each single-biomarker test, either in-house or through referral. The access levels are categorized as high (>75%), medium (50–75%), and low (<50%).
2. Timing: Timing refers to the average time from the availability of medicines to the availability of single-biomarker tests. It categorizes countries as “on time” (test available around the time of medicine launch) or “late” (a lag from medicine availability to test availability, i.e., >1 year).
3. Reimbursement: Reimbursement is based on the average proportion of tests reported to be covered by public reimbursement. It is categorized as high (>90%), medium (75–90%), or low (<75%).
4. Order Rate: The order rate is calculated based on the average order rates across focus biomarkers (PD-L1, EGFR, BRCA* (breast), BRCA (ovarian), NTRK, HER-2, ALK, MMR/MSI, KRAS /NRAS, BRAF, and ROS1). It is categorized as high (>75%), medium (50–75%), or low (<50%).

Score Calculation:

The score is calculated for each country based on the average of individual scores under single-biomarker test availability, single-biomarker test timing, single-biomarker test reimbursement, and single-biomarker test order rate. Performance on each individual score is rated as high (numerical score = 3), medium (numerical score = 2), or low (numerical score = 1), according to the thresholds mentioned above.

Score Categories:

High: Countries with a score ranging from 2.5 to 3.0;
Medium: Countries with a score in the range of 1.5 to <2.5;
Low: Countries with a score in the range of 1.0 to <1.5.

### 2.3. Statistical Analysis

Correlation is a statistical measure that describes the extent to which two variables change together. It quantifies the relationship between two sets of data; with changes in the other variable, it means that changes in one variable are associated with changes in the other variable. Correlation does not imply causation, meaning that just because two variables are correlated does not mean that one causes the other.

Correlation is typically measured using a correlation coefficient, the most common one being the Pearson correlation coefficient (also known as Pearson’s r). The Pearson correlation coefficient measures the linear relationship between two continuous variables, ranging from −1 to 1. A correlation coefficient of 1 indicates a perfect positive correlation, meaning that as one variable increases; the other also increases linearly. A correlation coefficient of −1 indicates a perfect negative correlation, meaning that as one variable increases, the other decreases linearly. A correlation coefficient of 0 suggests no linear correlation between the variables. Correlations were performed in the research priorities between different cancer types and between different countries. For each country, data on a given research pillar, as an aggregate form of the measures for a particular set of research priorities, were matched with data on a given biomarker test parameter. The Excel Data Analysis Toolpak was then used to perform correlations between research pillars and biomarker test parameters across all countries [21].

Ranking involves ordering data points from the highest to the lowest based on specific criteria that “Measure” values for each cancer type or country based on the responses. The highest value is assigned a rank of 1; the second-highest is assigned a rank of 2; and so on. If multiple data points have the same value, they receive the same rank, and the next rank is skipped.

The percentile of the data value from a set of data values is a statistical measure that gives the percentage of data values that fall below a given data value.

Percentiles for the values in a given data set can be calculated using the formula:

n = (P/100) × N

where N = number of values in the data set, P = percentile, and n = ordinal rank of a given value (with the values in the data set sorted from smallest to largest). Ranking and percentiles were used to compare cancer research priorities between countries [22].

## 3. Results

### 3.1. Research Priorities Based on Patients’ Responses—Countrywise

#### 3.1.1. Correlation

The following correlation table (Table 3) illustrates the degree of similarity or collaboration in research priorities related to cancer or related healthcare based on the defined measures (Table 2) among European countries. 

France and Italy, as well as Italy and Portugal, exhibit high positive correlations in their research priorities, suggesting a strong alignment in their approaches. Moderate positive correlations are observed between France and Germany, Greece and Italy, Hungary and Italy, the Netherlands and Portugal, and Portugal and Spain, indicating a moderate level of research synchronization in these countries. However, most other country pairings display lower correlation values, implying less similarity or collaboration.

In the context of cancer research, higher positive correlations signify a greater degree of alignment in research priorities among the involved countries. This alignment could lead to more fruitful collaborations, enabling researchers and patients to benefit from a broader pool of knowledge, diverse perspectives, and potentially more effective treatment options. However, the specific measures or pillars require close inspection to yield a comprehensive and precise interpretation of these correlations.

#### 3.1.2. Rank and Percentile Analysis

**Figure 2:**
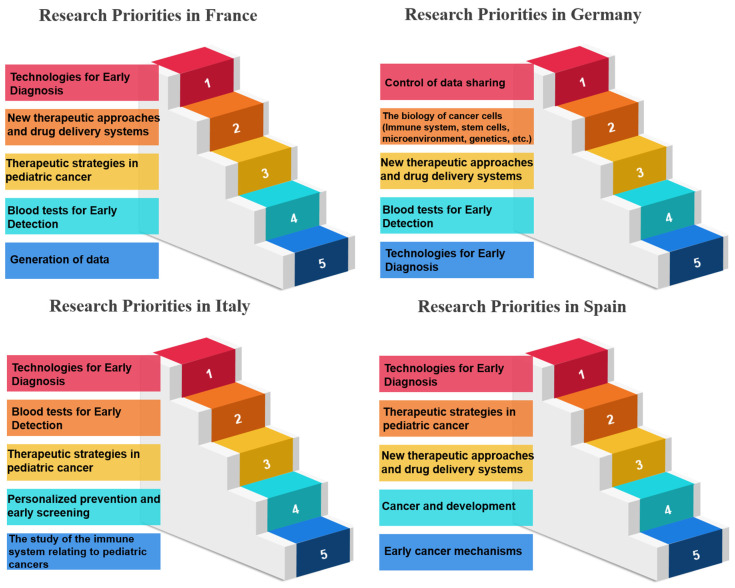
Research priorities in France, Germany, Italy and Spain based on survey responses.

**Figure 3:**
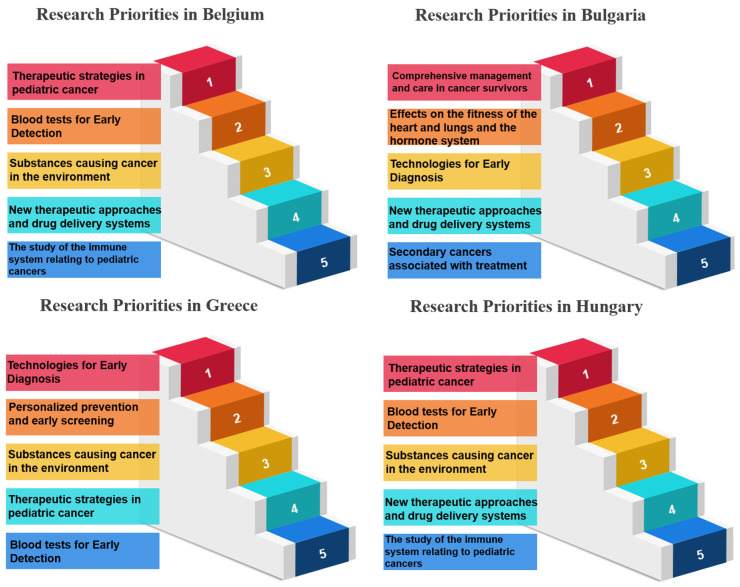
Research priorities in Belgium, Bulgaria, Greece and Hungary based on survey responses.

**Figure 4:**
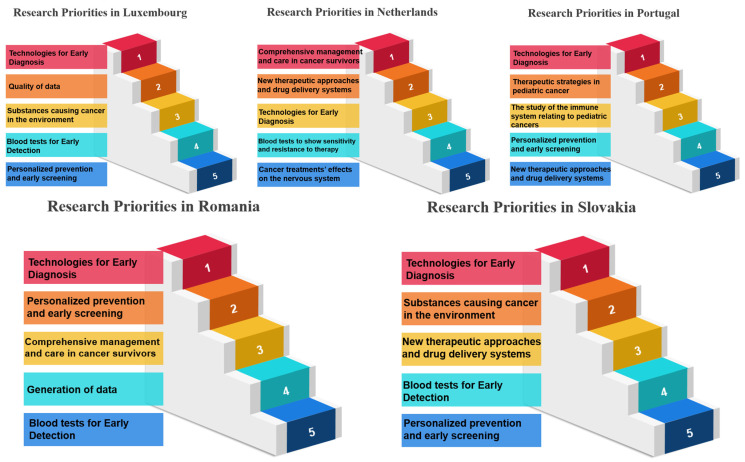
Research priorities in Luxembourg, Netherlands, Portugal, Romania and Slovakia based on survey responses.

In the realm of research priorities, certain measures stand out per country examined, and certain measures have garnered notable acclaim, signifying their importance in various scientific and developmental domains. Belgium, for instance, holds “Therapeutic Strategies in Pediatric Cancer” (Measure 17) in high regard, securing the top rank with an impressive 100.00%. Bulgaria values “Comprehensive Management and Care in Cancer Survivors” (Measure 30), achieving the same rank and percentage. France places significant emphasis on “Technologies for Early Diagnosis” (Measure 10), showcasing a commitment to research excellence with a perfect score. Germany accords utmost priority to “Blood tests to show sensitivity and resistance to therapy” (Measure 12), illustrating dedication to advancing this critical aspect of cancer research. Greece, on the other hand, emphasizes the “Prevention of Cancer” (Measure 5), highlighting a proactive stance in combatting cancer. Hungary focuses on “Cancer Heredity & Epigenetics” (Measure 6), aligning research initiatives with the genetic and epigenetic dimensions of cancer. Italy accentuates “Processes occurring before tumor development” (Measure 7), delving into the fundamental stages preceding the formation of tumors. Luxembourg places great importance on “Early Cancer Mechanisms” (Measure 8), showing dedication to understanding the initial mechanisms of cancer development. The Netherlands highlights “Blood tests for Early Detection” (Measure 9), prioritizing early diagnosis as a critical factor in cancer management. Portugal values “Technologies for Early Diagnosis” (Measure 10), reinforcing the significance of early detection tools. Romania directs its attention to “Personalized prevention and early screening” (Measure 11), aiming to tailor preventive measures and screenings. Slovakia emphasizes “Blood tests to show sensitivity and resistance to therapy” (Measure 12), recognizing the potential of blood-based diagnostics in guiding treatment choices. Last, Spain focuses on “The biology of cancer cells (Immune system, stem cells, microenvironment, genetics, etc.)” (Measure 13), highlighting the intricate biology of cancer cells and their surrounding environment (Table 4, Table 5 and Table 6) (Figure 2, Figure 3 and Figure 4). These distinct priorities across nations depict the diverse spectrum of research interests and allocations within scientific landscapes, providing a comprehensive view of their research emphases.

### 3.2. Research Priorities Based on Cancer Type

#### 3.2.1. Correlation Analysis

The investigators correlated research priorities (with respect to Measure 1, Measure 2, etc.) between cancer types independent of country. Correlations between the different types of cancer are reported in Table 7.

Breast Cancer:

Breast cancer has a correlation of approximately 0.80 with prostate cancer. This moderate positive correlation suggests that there may be some commonalities in research and treatment approaches between breast and prostate cancers, particularly in the context of hormonal influences, genetic factors, and targeted therapies.

Prostate Cancer:

For prostate cancer, one of the most prevalent cancers in men, research priorities often revolve around early detection methods, understanding hormonal influences, exploring targeted therapies, and managing potential side effects of treatment.

Lung Cancer:

Lung cancer shows moderate-to-strong positive correlations with other cancer types. For instance, it has a correlation of approximately 0.82 with colon cancer and 0.87 with breast cancer. This indicates potential commonalities in research and treatment strategies, such as exploring smoking-related risks, targeted therapies, and advancements in immunotherapies, which can be relevant for both lung and other cancer types.

Colon Cancer:

Colon cancer exhibits a strong positive correlation of approximately 0.79 with lung cancer and 0.75 with breast cancer. These correlations suggest potential shared aspects in research and treatment strategies, including investigations into risk factors such as diet and lifestyle, and personalized treatment approaches such as precision therapies and immunotherapies. 

Other Gastrointestinal Cancers:

The “Other Gastro Cancer” category, encompassing various gastrointestinal cancers’ correlation with prostate and breast cancer except colon cancer, exhibits moderate positive correlations among its members with prostate and breast cancer. This suggests that research priorities and treatment strategies for gastrointestinal cancers may share similarities among themselves and other cancer types, emphasizing areas such as risk factors, molecular pathways, and immunotherapeutic approaches relevant to this group of cancers (Table 7).

#### 3.2.2. Rank and Percentile Analysis

Breast Cancer:

Patients with breast cancer unanimously prioritize “Technologies for Early Diagnosis” (100.00% consensus) as their foremost research need. This high consensus reflects the critical importance of early detection in breast cancer, as it can significantly impact survival rates and treatment outcomes. The lowest-rated priority, “The Cell Biology of Aging and Cancer” (2.90%), underscores a potential mismatch between patients’ immediate needs and the focus of this research area. Breast cancer patients, like those with prostate cancer, seem to prioritize pragmatic solutions over more theoretical aspects of cancer biology.

Prostate Cancer:

Prostate cancer patients overwhelmingly expressed their top research priority as “Blood tests for Early Detection” (97.00% consensus). This indicates a strong demand for noninvasive and efficient methods to detect prostate cancer in its early stages, which is crucial for timely treatment and improved outcomes. On the other hand, the lowest-rated priority, “The Cell Biology of Aging and Cancer” (2.90%), reveals that patients may perceive this area of research as less directly applicable to their immediate concerns. This suggests that patients with prostate cancer are primarily focused on practical diagnostic and treatment solutions.

Lung Cancer:

Patients with lung cancer prioritized “Technologies for Early Diagnosis” (97.00%) as their top research priority. Early diagnosis in lung cancer is vital due to its often late-stage presentation, making early detection a key factor for improving patient outcomes. Similar to the other cancer types, “The Cell Biology of Aging and Cancer” remains at the bottom (2.90%), implying that lung cancer patients are also inclined toward practical research areas over more theoretical ones.

Colon Cancer:

Colon cancer patients present a distinct research priority by ranking “Use of Data” as their top concern (64.70%). This indicates that patients may value the utilization of data and research outcomes in shaping treatment approaches and decision making. Remarkably, “The Cell Biology of Aging and Cancer” is again rated as the lowest research priority (2.90%), reinforcing the trend that cancer patients often emphasize immediate and practical solutions. Colon cancer patients appear to value data-driven research as a means to improve their care and treatment decisions. 

**Figure 5:**
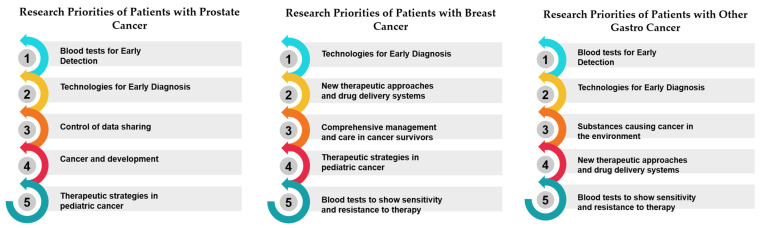

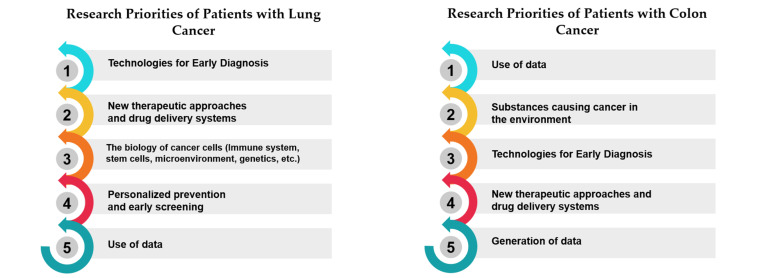
Research priorities of cancer patients in Europe based on survey responses.

Other Gastrointestinal (Gastro) Cancer:

Patients diagnosed with other gastrointestinal (Gastro) cancers also emphasize “Blood tests for Early Detection” as their top research priority, with 100.00% consensus. This highlights the universal demand for reliable and noninvasive methods of detecting gastrointestinal cancers at an early stage. As in the other cancer types, “The Cell Biology of Aging and Cancer” is rated the lowest (2.90%), suggesting that patients may prioritize research areas directly related to early diagnosis and effective treatments (Table 8) (Figure 5).

### 3.3. Correlation between Research Priorities and Access to Single Biomarkers

Table 9 correlates the research priorities among countries, broken down into the six foundational pillars, with the single-biomarker test parameters (see Section 2.3 for procedure used). A given test property may be influenced by survey recipients’ perceptions, as consumers and professionals, of the research importance of a given pillar.

Factors Influencing Cancer Development and Risk:

Availability (Correlation: −0.2121): Survey recipients’ prioritization of research on cancer development and risk is weakly correlated, and negatively so, with single-biomarker test availability. This suggests that as the availability of single-biomarker tests decreases, this pillar may have only a weak influence on cancer development and risk. Limited test access possibly but not necessarily hinders the identification and management of factors influencing cancer risk.

Timing (Correlation: −0.5258): With a strong negative correlation of −0.5258, timing delays significantly enhance the influence of this pillar, indicating significant delays in accessing these tests. This delay may impede timely risk assessment and intervention.

Reimbursement (Correlation: 0.0268): Correlations with reimbursement and order rate were very low, suggesting a minimal impact of these test parameters on survey recipients’ prioritization of research on cancer development and risk.

Order Rate (Correlation: −0.0661): The negative correlation of −0.0661 suggests that as the order rate decreases, the impact on this pillar slightly increases, reflecting lower demand for these tests and potentially a reduced focus on managing cancer risk factors.

Cancer Prevention and Early Detection:

Availability (Correlation: 0.2131): The positive correlation of 0.2131 suggests that as availability improves, this pillar’s influence on cancer prevention and early detection increases. Access to these tests likely contributes to more effective strategies for early detection and prevention.

Timing (Correlation: −0.1095): A slight negative correlation of −0.1095 indicates that moderate timing delays have a slight negative impact on this pillar. This implies that improving the timing of test availability is essential for enhancing early detection and prevention strategies.

Reimbursement (Correlation: 0.1150): The positive correlation of 0.1150 suggests that higher reimbursement coverage is associated with a slightly stronger influence on this pillar, highlighting the importance of financial support in this context.

Order Rate (Correlation: 0.1812): The strong positive correlation (0.1812) indicates that a higher order rate significantly enhances the influence of this pillar, signifying greater demand for tests in this context and reinforcing the importance of early detection.

Cancer Biology and Therapeutic Approaches:

Availability (Correlation: 0.3431): With a strong positive correlation of 0.3431, increasing the availability of single-biomarker tests significantly strengthens the influence of this pillar on cancer biology and therapeutic approaches. This indicates a solid foundation for advancing research and treatments.

Timing (Correlation: 0.0413): A slight positive correlation (0.0413) suggests that as timing improves, the influence on this pillar increases, although the effect is not as pronounced as availability. It underscores the importance of timely access.

Reimbursement (Correlation: 0.2765): The positive correlation of 0.2765 indicates that higher reimbursement coverage is associated with a considerably stronger influence on this pillar, underlining the role of financial support.

Order Rate (Correlation: 0.3218): A strong positive correlation (0.3218) suggests that a higher order rate significantly enhances the influence of this pillar, reflecting substantial demand for tests in this context and their crucial role in research and treatment.

Aging and its Intersections with Cancer:

Availability (Correlation: 0.0370): The positive correlation of 0.0370 suggests a slight increase in this pillar’s influence as availability improves, indicating that enhanced access to tests can have a modest impact on managing cancer in the context of aging.

Timing (Correlation: −0.1348): A slight negative correlation of −0.1348 implies that timing delays have a modest negative impact on this pillar. It reflects the complexity of addressing cancer in the context of aging and the need for timely access to tests.

Reimbursement (Correlation: −0.2231): A strong negative correlation (−0.2231) suggests that lower reimbursement coverage significantly enhances the influence of this pillar. Challenges in accessing tests related to aging and cancer are indicated.

Order Rate (Correlation: −0.0169): A slight negative correlation of −0.0169 indicates that as the order rate decreases, the influence on this pillar is only slightly impacted, reflecting a relatively stable focus on this aspect.

Cancer Complications and Survivorship

Availability (Correlation: −0.0493): The negative correlation of −0.0493 suggests that as availability decreases, the influence of this pillar on cancer complications and survivorship increases. Limited access may impede the management of complications and survivor support.

Timing (Correlation: −0.2651): With a strong negative correlation of −0.2651, timing delays have a negative impact on this pillar, potentially affecting the management of complications and survivorship in a substantial way.

Reimbursement (Correlation: −0.3642): A strong negative correlation (−0.3642) implies that lower reimbursement coverage significantly enhances the influence of this pillar, highlighting challenges in accessing related tests in this context.

Order Rate (Correlation: −0.1217): A strong negative correlation of −0.1217 suggests that as the order rate decreases, the impact on this pillar is significantly amplified, reflecting decreased demand for tests in this context and a need for greater focus and resources.

Data Generation and Utilization in Cancer Research:

Availability (Correlation: 0.1891): The positive correlation of 0.1891 indicates that as availability improves, this pillar’s influence on data generation and utilization in cancer research strengthens, highlighting the importance of access to data-related tests.

Timing (Correlation: −0.1553): A slight negative correlation of −0.1553 suggests that timing delays have a slight negative impact on this pillar’s influence, indicating that improved timing is essential for data-driven research.

Reimbursement (Correlation: 0.5672): A strong positive correlation (0.5672) implies that higher reimbursement significantly enhances the influence of this pillar, emphasizing the role of financial support in data-driven research.

Order Rate (Correlation: 0.2773): The strong positive correlation of 0.2773 suggests that a higher order rate significantly amplifies the impact of this pillar, indicating a strong demand for data-related tests in cancer research (Table 9).

## 4. Discussion

### 4.1. Research Priorities

The prioritization of specific measures in cancer research in the countries covered demonstrates a focused approach toward understanding, preventing, and treating cancer. Each country’s emphasis on particular measures aligns with their unique strengths, research infrastructure, and healthcare objectives. These research priorities collectively contribute to a broader understanding of cancer biology and intervention strategies.

Belgium’s emphasis on “Therapeutic Strategies in Pediatric Cancer” (Measure 17) underscores the critical need for advancements in pediatric oncology, aiming to develop effective therapies tailored for young patients. This aligns with global efforts to improve survival rates and reduce the long-term effects of cancer treatments in children [23].

Bulgaria’s focus on “Comprehensive Management and Care in Cancer Survivors” (Measure 30) reflects a growing recognition of the importance of survivorship care and the need to address the long-term physical, psychological, and social challenges faced by cancer survivors [24].

France’s emphasis on “Technologies for Early Diagnosis” (Measure 10) aligns with the global push for early detection technologies to improve cancer outcomes. Early diagnosis significantly impacts survival rates, and advancements in technologies are critical in achieving this goal [25].

Germany’s prioritization of “Blood tests to show sensitivity and resistance to therapy” (Measure 12) highlights the growing interest in liquid biopsy-based approaches to guide cancer treatment decisions. Liquid biopsies hold promise for noninvasive monitoring of treatment response and the detection of minimal residual disease [26].

Greece’s focus on “Prevention of Cancer” (Measure 5) indicates a proactive approach in combating cancer through preventive strategies. This aligns with global efforts to reduce cancer incidence by implementing preventive measures such as lifestyle modifications and vaccination programs [27].

Hungary’s attention to “Cancer Heredity & Epigenetics” (Measure 6) reflects the increasing recognition of the role of genetics and epigenetics in cancer susceptibility and progression. Understanding these factors is crucial for targeted therapies and personalized medicine [28].

Italy’s emphasis on “processes occurring before tumor development” (Measure 7) highlights the importance of studying the early stages of cancer progression, providing valuable insights into potential intervention points for effective cancer prevention [29].

Luxembourg’s focus on “Early Cancer Mechanisms” (Measure 8) indicates a commitment to understanding the initial steps in cancer development. Insight into early mechanisms can inform the development of early detection strategies and preventive interventions [30].

The Netherlands’ priority around “Blood tests for Early Detection” (Measure 9) aligns with the global interest in developing minimally invasive early detection methods. Blood-based tests offer a noninvasive and easily accessible approach to detect cancer at early, more treatable stages [31].

Portugal’s value for “Technologies for Early Diagnosis” (Measure 10) underscores the importance of technological advancements in cancer detection. Early diagnosis enables timely interventions, ultimately improving patient outcomes and reducing the burden on healthcare systems [32].

Romania’s attention to “Personalized prevention and early screening” (Measure 11) reflects the shift towards personalized medicine, tailoring preventive measures and screening strategies based on an individual’s genetic predisposition and risk factors [33].

Slovakia’s emphasis on “Blood tests to show sensitivity and resistance to therapy” (Measure 12) aligns with the global trend towards precision medicine, where understanding a patient’s unique response to therapy is crucial for optimizing treatment outcomes [34].

Spain’s focus on “The biology of cancer cells (Immune system, stem cells, microenvironment, genetics, etc.)” (Measure 13) highlights the intricate interplay between cancer biology, the immune system, and genetics. Targeting these aspects is essential for the development of effective cancer therapies [35].

Overall, the countries’ cancer research priorities demonstrate both shared themes and distinct focuses, reflecting diverse approaches shaped by individual strengths, healthcare objectives, and awareness of unique challenges. Commonalities, such as the emphasis on early detection technologies in France, Luxembourg, and the Netherlands, highlight the global recognition of the critical impact of timely diagnosis on cancer outcomes. Conversely, Belgium’s specialized focus on therapeutic strategies for pediatric cancer, Bulgaria’s attention to comprehensive survivorship care, and Greece’s proactive stance on cancer prevention illustrate unique national priorities. Italy’s interest in processes before tumor development, Hungary’s focus on genetics and epigenetics, and Spain’s intricate exploration of cancer biology, immune system interactions, and genetics further underscore the diversity in research emphases.

### 4.2. Correlations among Cancer Types and Research Priorities

#### 4.2.1. Correlations among Cancer Types

The correlations among different cancer types based on their research priorities provide valuable insights into potential commonalities and shared focus areas in oncology. Prostate cancer, as a prevalent cancer in men, understandably holds a perfect correlation with itself, highlighting the innate relationship between the research priorities for this specific cancer, typically revolving around early detection methods, hormonal influences, targeted therapies, and the management of treatment-related side effects. The implications of the prostate cancer research priorities are detailed in the paragraphs below.

Breast cancer, with a positive correlation with prostate cancer, suggests that there may be some commonalities in the research and treatment approaches between these two cancer types. This correlation likely stems from shared areas of interest, such as understanding hormonal influences, genetic factors, and the development of targeted therapies. Breast and prostate cancers often intersect in their focus on personalized treatment approaches [36].

The “Other Gastro” category, encompassing various gastrointestinal cancers, exhibits moderate positive correlations among its members with other cancer types. This finding implies that research priorities and treatment strategies have similarities with other cancers, emphasizing key areas such as risk factors, molecular pathways, and the exploration of immunotherapeutic approaches. The commonalities within this group of cancers may lead to collaborative research efforts that benefit patients with gastrointestinal malignancies.

Lung cancer, with moderate-to-strong positive correlations with other cancer types, reveals potential overlapping research and treatment strategies. These correlations suggest that research and treatment strategies, such as investigating smoking-related risks, developing targeted therapies, and advancing immunotherapies, can be relevant for both lung cancer and other cancer types. These intersections in research priorities may lead to cross-disciplinary collaboration and the sharing of insights.

Colon cancer, known for its prevalence and impact, exhibits a strong positive correlation with lung cancer and with breast cancer. These robust correlations indicate shared aspects in research and treatment strategies. Common areas of focus include investigations into risk factors such as diet and lifestyle, as well as personalized treatment approaches such as precision therapies and immunotherapies. These shared priorities emphasize the importance of cross-pollination of ideas and approaches among researchers and healthcare professionals to improve the outcomes for patients with colon cancer and related malignancies. This collaborative approach enhances the potential for innovative breakthroughs and advances in cancer treatment across multiple cancer types [37].

#### 4.2.2. Relation between Cancer Priorities and Access to Single-Biomarker Tests

The correlations between research priorities and access to single-biomarker tests offer crucial insights into the complex dynamics influencing various facets of cancer research and care. Within the scope of “Factors Influencing Cancer Development and Risk,” the negative correlation with availability implies that limited access to single-biomarker tests may impede the identification and management of factors influencing cancer risk, posing challenges in risk assessment and intervention. Furthermore, the significant negative correlation with timing underscores the vital importance of timely access to tests, as delays can obstruct risk assessment and early intervention, potentially resulting in adverse outcomes. Conversely, the correlation between reimbursement and this pillar suggests that increased financial support can slightly augment the capability to address cancer risk factors, highlighting the pivotal role of financial resources in this context.

The “Cancer Prevention and Early Detection” pillar reveals correlations between availability and reimbursement, indicating that improved availability and financial support can enhance the influence of this pillar. Access to tests contributes to more effective strategies for early detection and prevention, while higher reimbursement coverage fortifies the capacity to focus on these critical aspects of cancer care.

In “Cancer Biology and Therapeutic Approaches,” the robust correlation with availability implies that from the survey participants’ perspective increasing the availability of single-biomarker tests significantly strengthens the influence of this pillar. This suggests that a solid foundation for research and treatment is established when access to these tests is widespread. Additionally, the positive correlation with reimbursement underscores the importance in survey participants’ eyes of financial support in advancing cancer biology and therapeutic approaches, emphasizing the pivotal role of resources in shaping the direction of this field.

“Aging and its Intersections with Cancer” indicates that availability has a influence on this pillar. Improved access to tests can have a slight impact on managing cancer in the context of aging, suggesting potential benefits from making these tests more accessible to older populations. Conversely, the negative correlation with reimbursement highlights the challenges posed by lower financial support in addressing the unique aspects of aging and cancer.

In “Cancer Complications and Survivorship,” the negative correlation with availability implies that limited access to tests can increase the influence of this pillar, potentially due to difficulties in managing complications and providing survivor support. Timing delays have a significant negative impact, indicating that delays in accessing tests can affect the management of complications and survivorship. The negative correlation with reimbursement underscores the critical role of financial support in this context, as challenges in accessing related tests are indicated.

Lastly, in “Data Generation and Utilization in Cancer Research,” correlations with availability and reimbursement underscore the importance of access to data-related tests and financial support in strengthening the influence of this pillar. The improved availability of these tests enhances their impact on data generation and utilization, while higher reimbursement significantly amplifies the role of financial resources in data-driven research.

In summary, the correlations between research priorities and access to single-biomarker tests highlight the intricate interplay between these factors, providing insights into the critical role of timely access, financial support, and broader availability in shaping the landscape of cancer research, prevention, treatment, and survivorship. It is important to note that the pillar findings are based on data from survey recipients’ priorities, and caution should be exercised, especially considering potential weak-to-negligible between-country correlations [38].

### 4.3. Alignment with 13 Recommendations

The 13 recommendations for a comprehensive and inclusive approach to conquer cancer in Europe (Table 3) are aligned with the prioritization of specific measures in cancer research within various European countries. These recommendations provide a broader framework for understanding, preventing, and treating cancer, while the individualized measures in each country reflect their unique strengths, research infrastructure, and healthcare objectives. Here is how the recommendations are connected to the country- specific measures assessed in our study:

Belgium—Emphasis on “Therapeutic Strategies in Pediatric Cancer”: Recommendation 11 focuses on childhood cancers and improving outcomes for young patients, aligning with Belgium’s commitment to advancing pediatric oncology.

Bulgaria—Focus on “Comprehensive Management and Care in Cancer Survivors”: Recommendation 7 centers on improving the quality of life for cancer survivors and supporting long-term care, which corresponds to Bulgaria’s focus on survivorship care.

France—Emphasis on “Technologies for Early Diagnosis”: Recommendation 4 prioritizes optimizing screening programs and developing early detection methods, complementing France’s interest in early diagnosis technologies.

Germany—Prioritization of “Blood tests to show sensitivity and resistance to therapy”: Recommendation 6 advocates for an EU-wide research program on early diagnostics and minimally invasive treatment, which aligns with Germany’s interest in liquid biopsy-based approaches to guide cancer treatment decisions.

Greece—Focus on “Prevention of Cancer”: Recommendation 3 supports the development of effective cancer prevention strategies, in line with Greece’s proactive approach to reducing cancer incidence through preventive measures.

Hungary—Attention to “Cancer Heredity & Epigenetics”: Recommendation 5 aims to advance and implement personalized medicine approaches, emphasizing genetics and epigenetics, which is consistent with Hungary’s focus on cancer heredity and epigenetics.

Italy—Emphasis on “Processes occurring before tumor development”: Recommendation 5 highlights the importance of studying early cancer mechanisms and their role in cancer prevention, aligning with Italy’s priority around processes occurring before tumor development.

Luxembourg—Focus on “Early Cancer Mechanisms”: Recommendation 5, which emphasizes understanding early mechanisms in cancer development, complements Luxembourg’s commitment to studying early cancer mechanisms.

Netherlands—Priority on “Blood tests for Early Detection”: Recommendation 4 aligns with the Netherlands’ interest in blood-based tests for early cancer detection, as it also emphasizes developing minimally invasive early detection methods.

Portugal—Value for “Technologies for Early Diagnosis”: Recommendation 4 reinforces Portugal’s emphasis on technological advancements for early cancer diagnosis and the associated benefits for patient outcomes.

Romania—Attention to “Personalized prevention and early screening”: Recommendation 5, which promotes personalized prevention and early screening, corresponds with Romania’s focus on tailoring preventive measures and screening strategies based on individual risk factors.

Slovakia—Emphasis on “Blood tests to show sensitivity and resistance to therapy”: Recommendation 6 emphasizes the importance of understanding patient responses to therapy, which aligns with Slovakia’s focus on blood tests for therapy sensitivity and resistance.

Spain—Focus on “The biology of cancer cells (Immune system, stem cells, microenvironment, genetics, etc.)”: Recommendation 5 highlights the complex interplay between cancer biology, the immune system, and genetics, which corresponds to Spain’s emphasis on studying the biology of cancer cells and related factors.

In summary, the 13 recommendations provide a comprehensive and cohesive approach to cancer research and care in Europe, while each country’s specific measures contribute to these broader goals by leveraging their unique strengths and priorities in the fight against cancer [18].

## 5. Conclusions

This study provides evidence of the merits of collective effort in improving patient outcomes in cancer. Through an investigation of the attitudes, aspirations, and anxieties of patients and their families and other stakeholders in the cancer space, a more sensitive and tailored response to need and a more effective use of resources becomes possible. However, this effort depends on a shared commitment to advancing the understanding of cancer at the level of different patients, age groups, countries, and disease states. The data from the surveys in this study show how diverse research priorities reflect a nuanced understanding country-by-country of the multifaceted nature of cancer, opening the pathway to a clearer setting of goals and strategies for improving cancer care.

## Figures and Tables

**Table 1:** 13 recommendations [18].

Recommendation	Short Description
1. Launch UNCAN.eu—a European initiative to understand cancer	UNCAN.eu would integrate innovative models and technologies with longitudinal patient data, samples, and biomarkers to identify and translate to patients.
2. Develop an EU-wide research program to identify (poly-)genic risk scores	The research program would promote the clinical validation of polygenic risk scores (PRS), educational activities on the clinical importance of polygenic risk to all citizens regardless of their age, and encourage public debate on their use and control.
3. Support the development and implementation of effective cancer prevention strategies and policies within Member States and the EU	The Mission Board proposes a research program to identify strategies for cancer prevention and provide up-to-date knowledge to EU institutions and countries.
4. Optimize existing screening programs and develop novel approaches for screening and early detection	The program will optimize existing screening programs, develop new approaches for early detection of cancers, and include individualized approaches to screening.
5. Advance and implement personalized medicine approaches for all cancer patients in Europe	To increase its effectiveness, this recommendation encourages the optimization, implementation, and scaling of personalized medicine approaches for cancer.
6. Develop an EU-wide research program on early diagnostics and minimally invasive treatment	Accurate diagnostic methods are important to detect tumors at an early stage, predict treatment response, and detect tumor regrowth.
7. Develop an EU-wide research program and policy support to improve the quality of life of cancer patients and survivors, family members and carers, and all persons with an increased risk of cancer	Supportive policies need to be developed to identify and monitor physical and mental health problems among patients and survivors.
8. Create a European Cancer Patient Digital Centre where cancer patients and survivors can deposit and share their data for personalized care	The proposal is to create the European Cancer Patient Digital Centre (ECPDC), a network where cancer patients and survivors can deposit their medical data in a standardized and ethical way.
9. Achieve cancer health equity in the EU across the continuum of the disease	Policy support and interventions are necessary to address existing inequities across and within Member States.
10. Set up a network of Comprehensive Cancer Infrastructures within and across all EU Member States to increase quality of research and care	EU citizens or cancer patients should have access to accredited Comprehensive Cancer Infrastructures (CCI) in their country (at least one CCI in each member state), albeit through a national access point to an accredited CCI in another country, if relevant.
11. Childhood cancers and cancers in adolescents and young adults: cure more and cure better	Across Europe, cancer is the leading cause of death in children over one year of age. This population of cancer patients is characterized by several types of rare cancers, unique to them, with specific epidemiological, biological, and clinical features.
12. Accelerate innovation and implementation of new technologies and create oncology-focused living labs to conquer cancer	The goal is to provide new ways for traditional and nontraditional innovators to contribute to cancer understanding, prevention, diagnosis and treatment, and quality life support.
13. Transform cancer culture, communication, and capacity building	It is proposed to develop a coherent set of cross-sectoral activities to enable citizens, providers (including nurses, primary and other clinical doctors), researchers, other stakeholders (e.g., policy makers, health insurers, employers and trade unions), and communities within all Members States to think about cancer and challenge the culture of cancer in all its dimensions.

**Table 2:** Measures and measure descriptions.

Measure ID	Pillar ID	Measure Name	Measure Description
1	1	Gut Microbiome and Dietary Impact	The last decade has brought us a greater understanding of the impact of our ‘diet’ on intestinal ‘microbiota’ (gut bacteria), and how changes in the ‘microbiota’ are associated with our health (cancer promotion and prevention).
2	1	Metabolic Health and Physical Activity Influence	Studies have shown that lifestyle behaviors may impact metabolism and cancer risk.
3	1	Prolonged Inflammatory Responses	Studies have shown that inflammation that becomes chronic or lasts for too long is often associated with the development and progression of cancer.
4	1	Environmental Carcinogenic Factors	Studies have shown that some environmental factors, called also carcinogens, increase the risk of developing cancer.
5	2	Cancer Risk Reduction Strategies	By the use of chemo treatments, vaccines such as the human papillomavirus (HPV) vaccine (the immune system), and preventive drugs for certain cancer types.
6	2	Genetic and Epigenetic Cancer Influences	Studies have shown that cancers develop due to the accumulation of genetic (changes in the DNA sequence, some of which may be inherited) and epigenetic (changes not affecting the DNA sequence but its activity, that are noninherited) alterations.
7	2	Pre-Tumor Progression Phases	The development of cancer is a multistep process in which normal cells gradually become malignant through the progressive accumulation of molecular alterations.
8	2	Initial Cancer Development Phases	Cancer is a disease caused when cells divide uncontrollably and cooperate with other cells in their local environment, while fostering tumor progression.
9	2	Hematological Biomarkers for Early Detection	Specific blood tests are designed to identify tumor (bio)markers that may be found in the blood when some cancers are present before showing symptoms or being detected through conventional imaging approaches.
10	2	Advanced Early Cancer Diagnostic Technologies	Numerous cancer-associated deaths occur from cancers for which we do not screen. To overcome this, new scalable and cost-effective technologies are developed to allow for the detection and diagnosis of cancers at an earlier stage when these are more responsive to treatments.
11	2	Tailored Cancer Risk Management and Early Screening	Everybody does not have the same risk of developing a cancer. Careful analysis of individual risk factors to adapt prevention and systematic screening to the risk level would increase the rate of early diagnosis.
12	2	Hematological Assays for Treatment Responsiveness and Resistance	In the past two decades, specific tests have been developed to customize the treatment plan for a cancer patient according to the sensitivity and resistance patterns that can be monitored by analyzing the patient’s blood.
13	3	Cancer Cell Biology and Immune Microenvironment	Studies have shown that not all cancer cells are created equal, and they have the capacity to remodel the cells around them. There are intrinsic differences in the proliferative and invasive capacity of cancer cells within the same patient. Immune cells in their environment also acquire specific properties.
14	3	Innovative Anti-Cancer Therapies and Drug Delivery Methods	The development of more specific anticancer drugs, new types of biological and immune-mediated therapies, novel combinations of therapies with diverse mechanisms of action, and advanced drug delivery systems to target cancer cells more specifically have the potential to improve cancer treatment for patients and reduce long-term effects.
15	3	Hereditary Factors and Epigenetic Mechanisms in Pediatric Oncology	The contribution of nongenetic factors and the influence of the tissue environment remain poorly understood.
16	3	Oncogenesis and Growth Phases	The causes of the molecular changes during development that lead to cancer in children are mostly unknown.
17	3	Therapeutic Approaches for Pediatric Cancers	What is effective for an adult with cancer might not work for a pediatric cancer patient. Therefore, specific strategies to treat pediatric and adolescent cancer patients are needed.
18	3	Immunological Aspects in Pediatric Cancer	The immune system of children and adolescents is different from that of an adult. The efficiency of immunotherapy might vary depending on the age of the patient, and this needs to be better understood.
19	3	Maternal Factors and Pediatric Cancer Association	Epidemiological studies have suggested an association between maternal risk factors or exposure to carcinogens during pregnancy and pediatric cancer incidence. However, the precise factors and mechanisms involved remain unexplored.
20	4	Aging Factors and Cancer Susceptibility	The incidence of most cancers increases with age as, for most adults, age is associated with chronic conditions, decreased efficacy of the immune system, cumulative exposure to risk factors (carcinogens), and tissue aging with cell senescence. These events are causally associated with cancer.
21	4	Cellular Senescence in Cancer Biology	Aging is a complex phenomenon caused by the time-dependent loss of physiological organism functions, including those that protect from cancer development.
22	4	Aging and Carcinogenesis Relationship	Studies have shown that mechanisms of ageing are also found to occur in carcinogenesis. There is a need to better understand what aging and cancer development have in common and where the two processes diverge.
23	4	Aging Impact on Cancer Treatments	Various studies support the hypothesis that cancer and/or cancer treatment is associated with accelerated biological aging. These factors are key determinants of survivorship along with the long-term impact of cancer therapy on the biological aging of an individual.
24	5	Personal Adverse Events and Concurrent Medical Conditions in Cancer	In older patients affected by cancer, it is key to consider not only the characteristics of the tumor but to also pursue an integral geriatric assessment to systematically investigate factors that determine the patients’ well-being. In this context, research suggests that we may be able to measure a biological age, which will be more precise than civil age to guide therapeutic choices when treating cancer.
25	5	Treatment-Related Secondary Neoplasms	Although it happens infrequently, patients may develop a secondary cancer as a result of the treatment received to treat the primary cancer.
26	5	Persistent Immunological Consequences of Treatment	The effects of some cancer treatments can compromise properties of the immune system, rendering patients vulnerable to viral and bacterial infections or causing autoimmune conditions.
27	5	Reproductive Health Impact due to Cancer and Treatment	Cancer and its treatment can adversely impact reproductive function in both women and men. The effects of cancer treatment may lead to transient or permanent loss of fertility, sexual desire, and sexual function.
28	5	Cardiovascular, Respiratory, and Hormonal Health Impact due to Treatment	Both chemotherapy and radiation therapy to the chest can cause problems in the heart and lungs leading to potential cardiovascular and respiratory conditions that may be temporary or long-lasting.
29	5	Neurological Consequences of Cancer Treatments	Chemotherapy and radiation therapy can cause long-term side effects on the brain, spinal cord, and nerves, sometimes enhancing pain sensitivity.
30	5	Holistic Care for Cancer Survivors	For cancer survivors who are no longer in active treatment, their care needs include surveillance for recurrence, screening for the development of subsequent primary cancers, monitoring and intervention for the long-term and late physical and psychological effects of cancer and its treatment, and management of comorbid medical conditions, as well as routine preventive and primary care.
31	6	Data Generation in Oncological Research	The development of data that may guide more precise therapeutic choices and generate more efficacy in treating cancer patients.
32	6	Data Utilization for Informed Oncological Decision-making	Data whose analysis can inform precise disease diagnosis, their heterogeneity, the existence of constitutive predisposing factors, and the ability of the patient to support and favorably respond to a given therapy.
33	6	Data Collection and Analysis in Oncology	With the tools of data sciences, researchers can collect and analyze data to identify common mechanisms in a large series of patients with similar diseases. With data sciences, the higher the number of patients analyzed, the more precise the analysis.
34	6	Data Quality Assurance in Oncological Studies	The efficacy of data sciences requires data standardization and interoperability to be reused by multiple teams asking complementary questions.
35	6	Regulated Sharing of Patient Data for Oncology Research	Patient data sharing requires strict regulation to protect privacy (anonymization). While such regulation is mandatory, it must also be organized in a manner that favors rather than prevents patient data sharing at the European level to support cancer research.

**Table 3:** Correlation table depicting the extent of similarity or collaboration in research priorities concerning cancer or related healthcare across European countries.

	BE	BG	FR	DE	GR	HU	IT	LU	NL	PT	RO	SK	ES
BE	1.00												
BG	0.58 *^,#^	1.00											
FR	0.78	0.62	1.00										
DE	0.73	0.56	0.86	1.00									
GR	0.59	0.57	0.74	0.62	1.00								
HU	0.67	0.72	0.66	0.69	0.73	1.00							
IT	0.84	0.67	0.79	0.73	0.79	0.80	1.00						
LU	0.75	0.48	0.85	0.77	0.62	0.50	0.77	1.00					
NL	0.69	0.63	0.81	0.69	0.53	0.65	0.67	0.64	1.00				
PT	0.78	0.70	0.80	0.79	0.72	0.83	0.91	0.70	0.73	1.00			
RO	0.60	0.63	0.54	0.50	0.57	0.73	0.68	0.46	0.53	0.62	1.00		
SK	0.80	0.55	0.82	0.81	0.67	0.62	0.79	0.79	0.69	0.71	0.64	1.00	
ES	0.79	0.47	0.75	0.75	0.66	0.75	0.88	0.75	0.65	0.85	0.68	0.76	1.00

* Belgium—BE, Bulgaria—BG, France—FR, Germany—DE, Greece—GR, Hungary—HU, Italy—IT, Luxembourg—LU, the Netherlands—NL, Portugal—PT, Romania—RO, Slovakia—SK, Spain—ES. 1–0.8 = very high positive correlation, 0.79–0.6 = high positive correlation, 0.59–0.4 = medium positive correlation, 0.39–0.2 = low positive correlation, 0.19–0 = very low positive correlation. ^#^ In this table, a high-to-low correlation indicates the extent of similarity in terms of research priorities among countries. Positive or negative correlations signify the direction of the correlation.

**Table 4:** Rank and percentile of the top and least prioritized areas of research in European countries (France, Germany, Italy, Spain).

Meas. *	FR **	^#^ Rank	% ***	Meas.	DE **	Rank	%	Meas.	IT **	Rank	%	Meas.	ES **	Rank	%
10	68.4	1	100.0	35	72.6	1	100.0	10	62.4	1	100.0	10	78.2	1	100.0
14	64.7	2	94.1	13	69.5	2	94.1	9	56.5	2	97.0	17	77.3	2	97.0
17	64.7	2	94.1	14	69.5	2	94.1	17	55.0	3	94.1	9	73.9	3	94.1
9	64.0	4	91.1	9	68.4	4	88.2	11	53.0	4	91.1	16	67.2	4	91.1
31	61.0	5	85.2	10	68.4	4	88.2	18	52.1	5	88.2	8	66.4	5	88.2
3	26.5	31	8.8	19	36.8	31	11.7	23	32.5	31	11.7	22	39.5	31	11.7
22	26.5	31	8.8	1	33.7	32	8.8	1	30.5	32	8.8	1	38.7	32	8.8
1	25.0	33	5.8	27	32.6	33	5.8	20	29.6	33	5.8	2	38.7	32	5.8
20	23.5	34	2.9	20	31.6	34	2.9	22	28.4	34	2.9	27	37.8	34	2.9
21	21.3	35	0.0	21	30.5	35	0.0	21	27.8	35	0.0	23	37.0	35	0.0

* Measure, ** France—FR, Germany—DE, Italy—IT, Spain—ES, *** Percentage. Green spectrum = high priority, red spectrum = low priority. ^#^ In this table, the rank signifies the level of priority assigned to the corresponding measures, with green indicating the highest priority and red signifying the lowest priority among the measures in the participating countries. (Please refer to Table 2 for Measure names).

**Table 5:** Rank and percentile of the top and least prioritized areas of research in European countries (Belgium, Bulgaria, Greece, Hungary).

Meas *	BE **	^#^ Rank	% ***	Meas.	BG **	Rank	%	Meas.	GR **	Rank	%	Meas.	HU **	Rank	%
17	71.2	1	100.0	30	67.9	1	100.0	10	74.3	1	100.0	10	58.9	1	100.0
9	66.1	2	97.0	28	62.5	2	97.0	11	71.4	2	97.0	17	54.6	2	97.0
4	61.0	3	94.1	10	60.7	3	79.4	4	68.6	3	91.1	30	52.5	3	94.1
14	61.0	3	91.1	14	60.7	3	79.4	17	65.7	4	91.1	9	51.8	4	88.2
18	61.0	3	88.2	25	60.7	3	79.4	9	62.9	5	88.2	7	50.4	5	88.2
23	22.0	31	11.7	6	30.4	31	11.7	25	40.0	29	5.8	27	33.3	31	8.8
24	22.0	31	8.8	2	28.6	32	2.9	2	37.1	32	5.8	23	32.6	32	8.8
20	20.3	33	5.8	22	26.8	33	2.9	22	37.1	32	5.8	6	31.2	33	2.9
22	20.3	33	2.9	21	25.0	34	2.9	3	34.3	34	2.9	2	30.5	34	2.9
21	16.9	35	0.0	23	23.2	35	0.0	1	31.4	35	0.0	1	25.5	35	0.0

* Measure, ** Belgium—BE, Bulgaria—BG, Greece—GR, Hungary—HU, *** Percentage. Green spectrum = high priority, red spectrum = low priority. ^#^ In this table, the rank signifies the level of priority assigned to the corresponding measures, with green indicating the highest priority and red signifying the lowest priority among the measures in the participating countries. (Please refer to Table 2 for Measure names).

**Table 6:** Rank and percentile of the top and least prioritized areas of research in European countries (Luxembourg, the Netherlands, Portugal, Romania, Slovakia).

Meas. *	LU **	^#^ Rank	% ***	Meas.	NL **	Rank	%	Meas.	PT **	Rank	%	Meas.	RO **	Rank	%	Meas.	SK **	Rank	%
10	76.3	1	100.0	30	60.7	1	100.0	10	81.4	1	100.0	10	69.7	1	100.0	10	73.0	1	100.0
34	76.3	1	97.0	14	59.0	2	97.0	17	80.4	2	97.0	11	69.7	1	97.0	4	71.4	2	97.0
4	68.4	3	91.1	10	57.4	3	94.1	18	76.5	3	94.1	30	66.7	3	91.1	14	68.3	3	94.1
9	68.4	3	91.1	12	57.4	3	91.1	11	73.5	4	91.1	31	66.7	3	91.1	9	65.1	4	91.1
11	68.4	3	79.4	29	52.5	5	88.2	14	73.5	4	88.2	9	63.6	5	79.4	11	61.9	5	88.2
27	31.6	31	8.8	22	27.9	28	11.7	2	44.1	31	11.7	22	48.5	28	8.8	27	31.7	29	11.7
21	28.9	32	8.8	23	27.9	28	5.8	3	44.1	31	5.8	27	48.5	28	8.8	21	30.2	32	5.8
22	28.9	32	2.9	27	27.9	28	5.8	20	43.1	33	5.8	2	45.5	33	2.9	24	30.2	32	5.8
23	28.9	32	2.9	20	26.2	34	2.9	21	43.1	33	2.9	35	45.5	33	2.9	22	25.4	34	2.9
20	23.7	35	0.0	24	21.3	35	0.0	1	41.2	35	0.0	23	42.4	35	0.0	23	17.5	35	0.0

* Measure, ** Luxembourg—LU, the Netherlands—NL, Portugal—PT, Romania—RO, Slovakia—SK, *** Percentage. Green spectrum = high priority, red spectrum = low priority. ^#^ In this table, the rank signifies the level of priority assigned to the corresponding measures, with green indicating the highest priority and red signifying the lowest priority among the measures in the participating countries. (Please refer to Table 2 for Measure names).

**Table 7:** Correlations between different cancer types.

	PC	BC	OGC	LC	CC
PC	1				
BC	0.80 *^,#^	1			
OGC	0.70	0.77	1		
LC	0.69	0.87	0.82	1	
CC	0.79	0.79	0.64	0.75	1

Prostate Cancer = PC, Breast Cancer = BC, Other Gastro Cancer = OGC, Lung Cancer = LC, Colon Cancer = CC. * 1–0.8 = very high positive correlation, 0.79–0.6 = high positive correlation, 0.59–0.4 = medium positive correlation, 0.39–0.2 = low positive correlation, 0.19–0 = very low positive correlation. ^#^ In this table, a high-to-low correlation indicates the extent of similarity in terms of research priorities among countries. Meanwhile, positive or negative correlations signify the direction of the correlation.

**Table 8:** Rank and percentile of the top and least prioritized areas of research based on different cancer types.

Meas *	PC **	^#^ Rank	% ***	Meas	BC	Rank	%	Meas	OGC	Rank	%	Meas	LC	Rank	%	Meas	CC	Rank	%
9	64.2	1	1.00	10	62.3	1	1.00	9	67.7	1	1.00	10	78.7	1	1.00	32	70.0	1	1.00
10	64.2	1	0.97	14	57.1	2	0.94	10	64.5	2	0.97	14	76.0	2	0.97	4	66.0	2	0.97
35	62.3	3	0.94	30	57.1	2	0.94	4	58.1	3	0.94	13	73.3	3	0.94	10	64.0	3	0.85
16	60.4	4	0.88	17	56.1	4	0.91	14	54.8	4	0.91	11	69.3	4	0.91	14	64.0	3	0.85
17	60.4	4	0.88	12	55.5	5	0.88	12	51.6	5	0.88	32	68.0	5	0.88	31	64.0	3	0.85
20	30.2	31	0.12	23	33.7	31	0.12	29	32.3	27	0.12	20	33.3	31	0.09	1	34.0	31	0.09
23	28.3	32	0.06	1	32.5	32	0.06	21	29.0	32	0.09	22	33.3	31	0.09	2	34.0	31	0.09
24	28.3	32	0.06	20	32.5	32	0.06	27	25.8	33	0.06	2	32.0	33	0.06	20	32.0	33	0.03
3	26.4	34	0.03	21	32.2	34	0.03	1	22.6	34	0.00	27	30.7	34	0.03	22	32.0	33	0.03
21	24.5	35	0.00	22	31.3	35	0.00	23	22.6	34	0.00	1	22.7	35	0.00	21	30.0	35	0.00

* Measure, ** Prostate Cancer = PC, Breast Cancer = BC, Other Gastro Cancer = OGC, Lung Cancer = LC, Colon Cancer = CC, *** Percentage. Green spectrum = high priority, red spectrum = low priority. ^#^ In this table, the rank signifies the level of priority assigned to the corresponding measures, with green indicating the highest priority and red signifying the lowest priority among the measures in the participating countries. (Please refer to Table 2 for Measure names).

**Table 9:** Correlations between research priorities and access to single biomarkers.

Pillars	Availability	Timing	Reimbursement	Order Rate
Factors Influencing Cancer Development and Risk	−0.21 *^,#^	−0.52	0.026	−0.06
Cancer Prevention and Early Detection	0.21	−0.10	0.11	0.18
Cancer Biology and Therapeutic Approaches	0.34	0.04	0.27	0.32
Aging and its Intersections with Cancer	0.03	−0.13	−0.22	−0.01
Cancer Complications and Survivorship	−0.04	−0.26	−0.36	−0.12
Data Generation and Utilization in Cancer Research	0.18	−0.15	0.56	0.27

* ±0.19 to 0: very low correlation (on latter side can be negligible), ±0.39 to 0.2: low correlation, ±0.59 to 0.4: medium or moderate correlation, ±0.79 to 0.6: high correlation, ±1 to 0.8: very high correlation. ^#^ In this table, a high-to-low correlation indicates the extent to which pillar research priorities correlate with single biomarker test parameters among countries. Meanwhile, positive or negative correlations signify the direction of the correlation.

## Data Availability

All data generated or analyzed during this study are included in this published article.

## Supplementary Materials

The following supporting information can be downloaded at: https://www.mdpi.com/article/10.3390/healthcare12020259/s1, Survey S1: Survey questions for patients and citizens.
